# The Effects of Humor in the Media: A Critical Review of Experimental Research

**DOI:** 10.3390/bs16081414

**Published:** 2026-08-18

**Authors:** Nathan Miczo, Danyang Zhao

**Affiliations:** School of Communication, Media, and Experience Industries, Western Illinois University, Macomb, IL 61455, USA; d-zhao@wiu.edu

**Keywords:** humor effects, media effects, review

## Abstract

Humor has long been of interest to media effects scholars. As meta-analyses and systematic reviews accumulate, there is a need to continue scrutinizing the means of knowledge production. The purpose of this review was to investigate how humor was examined in experimental media effects research. In particular, we focused on how humor stimuli were constructed and how humor responses were measured. Based on a review of 47 published articles, we concluded that most studies examined herein did not manipulate humor message attributes, but rather employed a simple comparison between a piece of humorous content against a piece of non-humor content. Additionally, while most of the studies were guided by theory, only a minority of them included a humor theory; most of the studies used a measure of perceived humor for the manipulation check; the majority of studies used the humor response variable solely for the manipulation check; and only a few studies used the humor measure as a dependent variable. Recommendations are advanced to continue refining our understanding of the effects of humor in the media.

## 1. The Effects of Humor in the Media: A Critical Review of Experimental Research

Media, both traditional and new, are pervasive in modern societies. One prominent function of media is entertainment (Zillmann & Bryant, 1994), often served through content that is humorous. Humor has been “ever present in the mass media” (Kinsey, 1993/1994, p. 50) since its earliest days, a fact that has not gone unnoticed by humor researchers. Cantor (1977), for example, conducted a content analysis of humor on prime-time television and in cartoon magazines. Gruner (1976/1996) examined the role of humor as a tool for persuasion in mass communication, concluding that it might garner attention but was not effective in moving audiences. Additionally, humor in advertising has been the focus of voluminous research over the years, including a number of meta-analyses (e.g., Eisend, 2009; Kaur et al., 2022; Karpinska-Krakowiak et al., 2025).

Interest in the effects of humor has expanded beyond advertising, however, to encompass the full range of media. Accordingly, there has been a growth in the number of meta-analyses and systematic reviews examining the effects of mediated humor. In addition to persuasion more generally (Walter et al., 2018), the persuasive effects of satire have been explored meta-analytically by Dobmeier et al. (2023) and Burgers and Brugman (2022). A systematic review of social media humor in the COVID-19 pandemic was conducted by Alkaraki et al. (2024). Kaltenbacher and Drews (2020) provided a systematic narrative review of humor’s role in environmental communications. Becker (2020) reviewed research on political satire, offering various theoretical frameworks that might expand the understanding of satire’s effects.

Meta-analyses and systematic reviews are a starting point for theory building (Perloff, 2013). However, they are also a starting point for method building. That is, knowledge and understanding advance through the interplay of theory and methodological practice. Saucier and Walter’s (2021) meta-analysis, in particular, is relevant to this interplay. Citing the neglect of intrinsic message features, they included 80 studies, examining the methodological factors evident in published experiments aiming to enhance our understanding of what makes stimuli funny. Two of their results are highlighted here. First, they found a strong, positive relationship between exposure to humorous stimuli and measures of perceived humor. However, this association was subject to wide variability, leaving open further exploration of stimulus properties. Second, studies guided by mechanisms proposed by the “big 3” humor theories (described below) were more effective than those without such guidance, though no differences were found between the theoretical mechanisms. They concluded that more theory building is needed. This conclusion is echoed by Limbu et al. (2026) in their systematic review of humor in health communication messages. Thus, the purpose of this critical review is to examine the means of knowledge production. We do this by exploring the stimulus properties used in experimental research and the kinds of theories used to guide those studies. We focus upon experiments because that is the method where the selection and construction of humor stimuli are under the control of researchers.

This effort is guided by a framework developed by O’Keefe (2003) and Tao and Bucy (2007). Within this framework, a distinction is drawn between media (or message) attributes and psychological states. Message attributes are the variations defined by operational definitions concerning the properties of stimuli (i.e., the specific design features distinguishing one message from another). In an experiment, these variations are constructed by the researcher and should follow closely from a theoretical or conceptual perspective. Psychological states are variations in participant outcomes arising from exposure to stimuli (e.g., subjective feelings states, cognitive appraisals). These outcomes could be used to confirm the successful manipulation of message attributes, or they could be of interest as either intervening variables (i.e., mediators or moderators) or as outcomes in their own right. Given these diverse options, the second purpose of this review is to examine the psychological states measured in media effects-humor studies and how they are incorporated into study designs.

In order to achieve the purposes of this study, we will first examine varying approaches to conceptualizing humorous message attributes. Then, we will outline a model of the humor response process (the psychological state most relevant to humor stimuli). Following that, we will present the results of a review of 47 humor–media effects experiments. The discussion section highlights recommendations for more nuanced approaches to the study of humor and media effects.

## 2. Humor and Message Attributes

There are many different ways of examining humor attributes, only a few of which are examined here.

The “Big 3” Humor Theories. We begin with the “big three” humor approaches (i.e., superiority, relief, and incongruity theories) (Rancer & Graham, 2012). Superiority theory involves “winner and losers” (Gruner, 1997). That is, humor involves social comparison processes whereby our laughter signals our feelings of elevation relative to the derogation of some target. Media humor often involves stereotypical depictions deployed, for example, in situational comedies (sitcoms) with stock characters or by comedians making jokes about public figures. These forms of disparagement humor often invoke group identities, reinforcing ingroup/outgroup dynamics (Martineau, 1972; Miczo, 2022). Both prejudiced norm theory (PNT; Ford & Ferguson, 2004) and the disposition theory of humor (DTH; Zillmann & Cantor, 1976/1996) provide propositions useful in the selection of humorous messages that manipulate stereotypes to arouse appropriate states in message recipients.

Relief (or release) theories focus upon laughter as a way to release tensions and inhibitions (Carrell, 2008; Morreall, 2009). Though early versions relied on a defunct hydraulic model of human emotions, the ideas persist today in the form of coping humor and humor as liberatory. Given that the goal of this type of humor is to create relaxation and positive affect, the intention of the message should be to alleviate existing anxieties or to arouse anxieties and then disperse them. When the goal is to challenge inhibitory beliefs or practices, messages might offer commentary on current practices or raise issues and then humorously undermine them.

Incongruity theory may be the broadest of the meta-theories, in part, because incongruity is taken by many to be a defining feature of all humor. Some versions of the theory argue that becoming aware of a discrepancy between what is expected (e.g., a schema) in a situation and perceptions of the actual situation is sufficient for humor. Others argue that humor arises from the attempt to resolve the incongruity. Suls’s (1972) model argued that humor involved a two-step process of comprehending an incongruity and then resolving it. Many forms of media humor involve a set-up and a punchline. Often, the set-up is a factual statement or a depiction of something “in reality.” The punchline presents the incongruity, requiring the receiver to “double back” and do the inferential work of making sense of the humor.

Collectively, these three theories offer what van der Wal et al. (2020) refer to as an “intertheoretical-perspective” (p. 480). Each one directs attention to a part of the humor experience, while claiming to completely explain humor and laughter. They are, then, more akin to meta-perspectives; they are non-falsifiable accounts that could be used to analyze and explain any humor stimuli. As such, they provide insights into why we laugh but are insufficient to provide much guidance for examining the attributes of humor messages.

Incongruity as Design Feature. Returning to the notion that incongruity is a feature of all humorous messages, any piece of humor should be analyzable in terms of its incongruities. Three positions are presented here. First, Veatch (1998) argued that humor arises from the simultaneous juxtaposition of a normal view of the situation (N) (i.e., the way the world should be) and a violation of that normal view (V). The violations are moral in the sense that they have to involve principles about the right way of doing things or appropriate behavioral conduct. Violations, then, cannot be so slight they are unnoticed, or so extreme that they are not entertained simultaneously with the normal view. Using Veatch’s theory to design humorous messages would require identifying both N and V.

The general theory of verbal humor (Attardo, 2001; GTVH) builds upon Raskin’s (1985) earlier semantic script theory of humor, which posits that a “joke-carrying text” must (1) be fully or partially compatible with two different scripts, and (2) the two scripts must be opposite. The GTVH expands upon this definition by including six different knowledge resources. The first two have received the most attention: (1) the script opposition (SO) as defined by Raskin; (2) the logical mechanism (LM), which is “the attempt by the text to explain away the incongruity by justifying it (once more, playfully and non-seriously)” (Attardo, 2017, p. 133), that is, the LM provides the (often partial) resolution of the humor; (3) language refers to the linguistic description of the humor; (4) narrative strategy refers to the organization of the text with respect to, for example, the placement of humor; (5) target is an optional element insofar as humor may or may not include a “butt;” and, (6) situation refers to the understanding necessary to accepting the “joke world” rather than the situation in which a particular bit of humor is produced. Though the theory privileges verbal humor and short texts, there have been some attempts to expand its application (Gérin, 2013; Tsakona, 2009).

Oring (2003) has been critical of the formalism of approaches such as the GTVH. His notion of appropriate incongruity is that humor involves “the perception of an appropriate relationship between categories that would ordinarily be regarded as incongruous” (p. 1). Consequently, humor does not require a two-step process of recognizing an incongruity and then resolving it, nor does it require a sequential ordering of those processes. What separates humor from other forms of “appropriate incongruity” such as metaphor is the realization that the connection between the categories is spurious, that it “violates logic, the sense of what we know to be true, or the sense of what traditional behaviors or expressions are supposed to do or mean” (p. 6). Less formal than the GTVH, Oring’s conceptualization also does not require the kinds of moral commitments advocated by Veatch.

In sum, from an incongruity perspective, humorous message present, sequentially or simultaneously, a normal situation (factual, corresponding to reality) and a relevantly incongruous situation (non-factual, non-correspondent with reality), amenable to a sense-making process. Theoretically, any humorous stimuli should be analyzable in terms of its script oppositions and the sense-making involved in “getting” the humor. From a design perspective, researchers should be able to identify the dual situations and the logics that connect them.

Humor genres. Though genre as a concept can be difficult to define (Coutinho & Miranda, 2009), according to Askehave and Nielsen (2005), genres consist of three elements: (1) a shared communicative purpose, (2) common structures and formats, and (3) strategies of content and style. Common genres of humor, such as disaster jokes, political comedy, and television sitcoms, reflect all three elements. Rather than attempt a list of content/topic areas, Tsakona (2017) presents a continuum of humor genres: (1) humor as an obligatory feature of the genre (e.g., jokes, comedies, sitcoms, Internet memes)—the primary communicative purpose of these forms is humorous entertainment; (2) humor as an optional but expected feature of the genre (e.g., conversational interaction, online posts, advertisements)—humor is not obligatory, but is frequently realized within the form; (3) humor as an optional but unexpected feature of the genre (e.g., business negotiations, political speeches, school textbooks)—humor in these forms should be more surprising, but not considered completely out of bounds; and (4) humor as an untypical feature of the genre such that it hardly ever (or never) occurs (e.g., court decisions, funeral speeches, religious texts)—although humor is possible in these forms, it will rarely occur and will therefore likely be highly unexpected.

Tsakona’s (2017) continuum is relevant to a consideration of message attributes because the choices that researchers make intersect with the expectations of participants. For example, in an experiment where one group of participants is presented with a sitcom where the humor has been left in, and another group is presented with the sitcom where the humor has been removed, each humorous example can be analyzed in terms of its incongruities, but the sitcom genre also primes both groups to expect the presence of humor (i.e., it is obligatory to this form). Thus, the reaction in the group experiencing no humor may also be influenced by the violation of their expectations. When designing humorous messages, researchers should be able to specify the degree to which their manipulations contain obligatory humor.

Contextualization cues. Many humor scholars have argued that humor is a form of play. When producing humor, therefore, it is important to incorporate verbal, nonverbal, or situational cues that frame a message as nonserious. These elements are known as play frames or contextualization cues (Coates, 2007). Of course, genres themselves can be framing devices insofar as they prime audiences to switch to a nonserious, playful mindset, taking Oring’s (2003) “spurious logic” in stride. However, there are also more specific cues that can be manipulated as message attributes. One common technique, especially in conversational humor, involves joke-prefacing devices (Cashion et al., 1987). These are statements such as ‘Did you hear the one about…” or “I heard this joke today.” Such statements alert listeners that what follows is a humor attempt and should be evaluated accordingly.

Another contextualization cue is the presence of laughter. This is usually premised on the idea of laughter’s contagiousness (Chapman & Chapman, 1974). Laugh tracks are frequently added to sitcoms and some evidence suggests their effectiveness (Pradhan et al., 2021). Additionally, the presence of others who are laughing can affect our own mood and behavior. As a message attribute, laugh tracks can be added (or removed) and confederates can be used during co-viewing contexts.

Techniques. There are a number of attempts to classify types of humor stimuli into specific techniques or strategies for eliciting humor responses (Berger, 1993). Table 1 presents three typological approaches: Buijzen and Valkenburg’s (2004) analysis of audio-visual material, van der Wal et al.’s (2020) content analysis of tween programs, and Ruch et al.’s (2018) comic styles markers approach. As has been pointed out by many commentators (e.g., Raskin, 1985, regarding humor typologies), typologies differ in terms of their intentions, definitions, and lists of specific techniques. Accordingly, there is no typological approach that can comprehensively account for all forms of humor. A typological approach might still be used if researchers are focusing upon a specific technique, or if they are comparing different forms of humor.

Affect. According to Monahan (1995), affect as a message design feature refers to the emotional valence of a message. Within the broader field of humor studies, there has been a growing recognition of positive and negative forms of humor (Cann et al., 2009). Positive forms of humor focus more on the “bonding” function of unification (Meyer, 2000), whereas negative forms focus more on the “biting” function of division (Meyer). This is reflected somewhat in the typologies insofar as van der Wal et al. (2020) and Ruch et al. (2018) found factors reflecting this distinction. That is, van der Wal et al.’s “fun” and “buffoonery” factors align with the “light styles” of Ruch et al., while “disparagement” aligns with the “dark styles.”

Although not as developed as some of the other approaches reviewed here, there may be merit in considering this distinction from the message attribute perspective. Monahan (1995) posited that positive affect messages are effective at getting attention and fostering good feelings within recipients. On the other hand, negative affect messages can stimulate the use of “more elaborate, detail-oriented, and analytical processing strategies” (p. 86). Thus, the outcomes of interest should align with the emotional tone of the humorous stimuli. One should not expect deep processing from slapstick or wordplay; on the other hand, both jokes that disparage some group based on sex, ethnicity, weight, etc., and political comedy that mocks public figures share a negative tone with the potential to foster more central processing.

Every study that manipulates humor is susceptible to analysis from multiple perspectives. That is, the humor stimuli used must employ incongruities, embedded within some genre, using some specific technique, and may or may not contain additional contextualization cues or emotional affect cues. As a preliminary step toward exploring humorous message attributes, we posed the following research question:

RQ1: What message attributes are used in humor-related manipulations?

Related to this is the issue of whether or not these attributes are guided by theoretical considerations. Saucier and Walter (2021) found that using any of the “big 3” theoretical mechanisms was more effective than not using any of them, but their definitions of the mechanisms were broad and inductively defined by coding the empirical stimuli for the meta-analysis. While it is claimed that more theory building is needed (Saucier & Walter, 2021), in order to evaluate that claim we need to know the range of theories and concepts that have been used to guide the construction of experimental designs where manipulation of message attributes is a salient concern. This is the first step in understanding the correspondence between theories used and message attributes chosen. The second research question reflects this interest:

RQ2: What theories and concepts are used in humor–media effects studies?

## 3. Humor and Psychological States

To say that humor is subjective is trivial, since ultimately, the reception and interpretation of any message is a subjective act. In the same way that theoretical progress cannot be made if the attributes of humorous messages are not carefully attended to, such progress will be hampered if the range of psychological states potentially evocable by humor is not carefully delineated. Saucier and Walter (2021) report that “funny,” “humorous,” “amusing,” and “entertaining,” were among the most commonly used terms for perceived humor in their meta-analysis of 80 studies. These terms may or may not be semantically equivalent. More importantly, the terms may mask more than they reveal. At issue is the range of possible responses when a participant is presented with a humor stimulus.

Carrell (1997) distinguished between joke competence and humor competence. Joke competence refers to the ability of native speakers (members of a shared culture) to recognize a message as a joke. She argued this was a fairly static ability as it was rooted in cultural or group conventions of shared language. Humor competence was the dynamic judgment of a joke’s funniness, resulting in subsequent amusement (or not). Building on Carrell’s distinctions, Hay (2001) argued that there are many different ways of showing humor support, and that recognizing and understanding humor were separable processes. She specified a sequence of recognition, understanding, and appreciation, with appreciation being further distinguishable from agreement. As a result, laughter implicates appreciation, but need not signal agreement.

Examining cross-cultural interaction, Bell (2007) further complicated this developing model. That is, though recognition may be tightly linked to linguistic/cultural competence, understanding/comprehension can be a social activity affected by situational factors. Non- or partial understanding of a joke does not preclude appreciation of humor, and laughter may indicate recognition rather than appreciation or agreement.

Combining across these models, we can state that responses to humor can be analytically separated into a set of processes: (1) the recognition of a message as humor (strongly influenced by linguistic/cultural competence); (2) understanding/comprehension of the script oppositions/expectation violations/incongruities involved in the humor (depending heavily on shared knowledge and common ground); (3) appreciation of the humor (affected by any number of social and contextual factors); (4) agreement with the premises and/or implications of the message, or with the situational appropriateness of humor, or even with the appropriateness of this speaker uttering this humorous message in this context.

Looking back at Saucier and Walter (2021), it seems likely that most studies intend their measure of perceived humor to operate at the level of humor appreciation, defined as an affective reaction that is pleasant and enjoyable. There are problems with this. First, perceived humor as humor appreciation may be misused as a manipulation check. That is, the appropriate question when humorous stimuli are being compared to serious or “no humor” conditions involves humor recognition (e.g., “did the message include humor”). Items such as “The message was—not humorous/humorous” are, therefore, strategically ambiguous in conflating recognition and appreciation.

Second, the proposed link between a humor response and an outcome of interest may depend more on one process rather than another. To gain attention, recognition or appreciation may be the most essential mechanisms. For learning or knowledge acquisition, comprehension of the content or the script oppositions may be necessary. Attitude change or behavioral activation may require agreement with the premises or the implications of the humor stimuli. Moving beyond the use of perceived humor as a manipulation check, incorporating humor responses as mediators or moderators, will necessitate more nuanced measures of the relevant state. We expect that most studies will include a measure of “perceived humor” similar to the measures reported in Saucier and Walter (2021). However, it is necessary to update their analysis with additional humor–media effects studies.

RQ3: What psychological states are measured from humor-related manipulations?

The framework we described earlier posits that theoretical advancement depends on incorporating mediators and moderators. In the case of humor studies, the humor response itself may often be a mechanism through which “humor” exerts its effects. In other words, the finding of a strong relationship between a humor manipulation and “perceived humor” argues for more attention to what is meant by “perceived humor” and how that psychological state is the mechanism for the outcomes that are more typically of interest to media scholars (e.g., attention, recall, persuasion, learning). Accordingly, we pose a research question to allow us to explore these linkages:

RQ4: How are humor-related psychological states used in experiments?

## 4. Methods

We conducted a critical review to synthesize the evidence on our topic. The searches for articles were conducted between September 2025 and January 2026.

### 4.1. Data Sources and Search Strategy

An initial limited search was conducted by one of the authors on the Communication and Mass Media Complete database, Google Scholar, PsychINFO as well as in the journals European Journal of Humor Research and Israeli Journal of Humor Research using the key words search string “humor” AND “media effects” with a publication date from 2008 to 2026. The initial search involves title and abstract screening. Subsequent full text screening and a secondary search were conducted by the two authors. The secondary search was done in the flagship journal of media effects, Media Psychology, using the key word “humor” as well as in the PsycARTICLES database using the search string “humor” AND “media effects”. Then, one of the authors did another targeted search in the references list of some existing records. Finally, the two authors discovered a few additional articles by the same first author when discussing excluding one of the ineligible ones. The initial screening returned 40 articles.

After each search, the two authors further screened all records for both abstract and full-text level. Disagreements were discussed and resolved between the two raters. As a result, thirty-four records met the inclusion criteria.

An additional 16 articles were obtained based on feedback from two reviewers of the initial manuscript. Ultimately, from the total corpus of 50 articles, three were removed, two because it was discovered during coding that the studies were correlational in design, and one was a direct replication of a study already in the corpus. Thus, the total number of articles coded for this study was 47.

### 4.2. Inclusion and Exclusion Criteria

The eligibility criteria of the review can be found in Table 2. Articles were considered as eligible for inclusion if they examined the effects of humorous messages using experimental methods with the humorous content being the stimulus material in the experimental group. We excluded studies in advertising research due to the unique nature of advertising messages. We retrieved the following information for all eligible articles after initial screening: author(s), title, year, topic, message attributes, theory or concept used in the rationale, psychological state (i.e., measure of humor response to the stimuli), use of a manipulation check and whether or not a humor-related measure was used as a variable in the analyses (e.g., mediator). The data was coded in a Google Excel spreadsheet. Next, two authors independently evaluated half of the 47 articles to assess their eligibility for inclusion in this review.

### 4.3. Data Extraction and Study Variables

The authors iteratively developed and refined the codebook through discussions and revisions. An eligible sample of 47 studies were split in half and the two authors each independently coded twenty-five articles. Questions and borderline cases were addressed through discussions between the two authors. Following the completion of coding, the first author double checked the codes against the corpus.

### 4.4. Message Attributes

Based on our review of the literature, the following categories were developed to identify message attributes:

(1) Genre: one group is exposed to a humorous message or format; the other group is exposed to a serious version of the same message or a non-humorous message
(2) Contextualization cues: groups are exposed to the same humorous message but something else is manipulated to induce humor (e.g., laugh track, co-viewers, comments added to a social media message)
(3) Technique: a specific type of humor is manipulated (e.g., puns, wordplay); this could also include situations where one group is exposed to one type of humor and another group is exposed to a different type of humor (e.g., relevant vs. irrelevant humor)
(4) Script opposition (SO): study specifically manipulates the content of a humorous message so that two versions are created that differ in the punchline (or resolution of the humor)
(5) Emotional valence: study specifically manipulates the content of a humorous message so that two versions are created that differ in the emotional tone of the messages (i.e., one version is a positive humor message and the other is a negative humor message, or, different degrees of one or the other valence).

### 4.5. Use of Theory or Concept

The coding scheme used here was derived inductively. Initially, an attempt was made to use the “big 3” humor theories to group theories/concepts that were used to guide study designs. However, the attempt proved impracticable. Though some studies used a well-defined humor approach, such as disparagement humor, others were less developed or restricted to a specific concept (e.g., parody). Of particular note, several studies used a cognitive processing approach that can be applied to processing humorous messages but does not align with incongruity or incongruity-resolution approaches (usually considered the most “cognitive” of the big 3).

### 4.6. Psychological States (Humor Responses)

This code concerns a humor-related measure that was used following exposure to a humorous stimuli in an experimental design. Following Saucier and Walter (2021), we use “perceived humor” as the umbrella term for any measure that contains items such as “humorous,” “funny,” “amusing, “entertaining,” etc. The measure could be a single item or multiple items, Likert-type or semantic differential.

We also coded measures that referred to other parts of the humor response process, as long as the measure contained items related to the humor stimuli. For example, measures asking if participants remembered information from the message were excluded, but a measure asking if participants agreed with the content of the jokes was considered an instance of humor agreement.

### 4.7. Manipulation Check of Humor Stimuli

Manipulation checks of experimental stimuli help increase the scientific rigor of research and help eliminate threats to internal validity. We are interested in whether a study conducted a manipulation check of humorous content before or after the experimental procedure.

### 4.8. Indirect/Conditional Effects

We are interested in the mediators or moderators introduced in the studies. Therefore, we coded this variable with four categories: mediation, moderator, moderated mediation, and a combination of two or three of the previous categories.

## 5. Results

Table 3 presents the results of the article coding.

The first research question concerned message attributes, the particular operationalizations that were used in experimental designs. Of the 47 studies, 26 (55%) were coded in the genre category. That is, these studies compared a humor condition against a serious or a nonhumorous condition (or conditions). Six studies (12.77%) manipulated some type of contextualization cue, including a laugh track, co-viewers, or a framing message. Similarly, six studies (12.77%) were coded as manipulating a particular humor technique, or comparing two kinds of humor. Studies that manipulated a humor technique and also included a no-humor control group were considered a mixed category (8 studies, 17.02%). Finally, one study (0.02%) manipulated a contextualization cue and included a control group.

RQ2 focused upon the theories or concepts that were used to guide or frame the experimental designs. Though a variety of approaches were utilized, an attempt was made to create some larger categories by grouping related concepts. Accordingly, the largest category involved cognitive processing approaches (11, 23.04%), typically using some form of the elaboration likelihood model (Petty & Cacioppo, 1986). A set of categories was constructed using the “big 3” humor theories for guidance. Terms related to superiority theory (e.g., disparagement humor, satire, hostile humor) classified 9 studies (19.15%). The specific terms incongruity or incongruity-resolution classified 4 studies (0.09%). Concepts specific to relief or coping humor comprised 3 studies (0.06%). Specific humor theories were drawn upon as follows: disposition theory of humor (3, 0.06%), benign violations theory (2, 0.04%), and prejudiced norm theory (1, 0.02%). Additional concepts used with some frequency were parody, used 5 times (11%), laughter used 3 times (0.06%), and amusement used 3 times (0.06%). Finally, 5 studies (11%) were considered underdeveloped (i.e., justification for examining humor was based on the results of prior empirical studies).

RQ3 concerned “psychological states” assessed in response to exposure to humorous stimuli. Thirty-five (74.47%) used a measure of perceived humor. Three studies (0.06%) used a measure of humor recognition, including 2 that used Nabi et al.’s (2007) message discounting measure.^1^ Three studies (0.04%) included some measure of agreement with the content of the humor message. The following measures were used once each: comprehension, emotional experience, enjoyment, positive affect. Finally, 8 studies (17.02%) did not include a measure of psychological state.

The final research question (RQ4) considered how the humor response variables were used across studies. The measure was used as a manipulation check in 24 (51.06%) studies. Additionally, 14 (29.79%) studies included a pilot or pretest (though some studies included both a pilot/pretest and a manipulation check). Six (12.77%) studies used the psychological state variable as a dependent variable, while 17 (36.17%) used it as either mediator, moderator, or in moderated mediation.

## 6. Discussion

The purpose of this review was to investigate how humor was examined in experimental media effects research. Based on a review of 47 published articles, we concluded that most studies examined herein did not manipulate humor message attributes such as contextualization cues and humor technique in their experimental design, but rather employed a simple comparison between a piece of humorous content against a piece of non-humor content. Additionally, while most of the studies were guided by theory, only a minority of them included a humor theory. Moreover, most of the studies used a measure of perceived humor for the manipulation check. And lastly, the majority of studies used the humor response variable solely for the manipulation check. While just over a third of the studies used a humor response variable as a mediator or a moderator, only a few studies used it as a dependent variable.

As informed by various theoretical perspectives in humor studies, there are multiple aspects of humor that could contribute to its psychological effects. These include but are not limited to the specific technique used (e.g., puns, irony), the genre of the content (e.g., comedy, drama), the contextualization cues it contains (e.g., laughing track, sound effects), and the presence of any affective cues. Experimental designs that merely contrast humorous and non-humorous conditions are not taking into consideration these nuances and therefore limited in their ability to account for the underlying mechanisms driving any observed effects of humor. This also limits the generalization of findings pertaining to the humor concept being examined.

When it comes to theoretical guidance, we argue that the development and selection of humor stimuli need to be guided by theories of humor specific to the characteristics of such stimuli in order to advance our understanding of how a given aspect of a humorous stimulus makes it funny. Theory-guided humor stimuli selection can not only test or advance the theory to help generalize the relationships it predicts, but also contribute to a concerted effort to improve the operationalization of humor concepts in future studies. For example, Wilson et al.’s (2025) manipulation of coping humor in a cancer-based film was based on relief theory, with mirth as a mediator between condition and character identification and cancer anxiety. However, they also created a character coping humor scale to assess evaluations of the characters and the film overall. Such an approach allows for an expanded examination of how the humor stimuli exerted its impact.

Though the literature of humor suggests that humor response manifests in four distinct dimensions: recognizing the content as humorous, comprehending the humor, appreciating it, and agreeing with the underlying message it conveys, the manipulation check in most studies reviewed herein were done at the humor appreciation level without assessing the other three dimensions of responses. This therefore introduces considerable ambiguity in interpreting the results of a single-dimension manipulation check and potentially obscuring the factors responsible for the effectiveness (or ineffectiveness) of the stimuli and complicating any causal inferences regarding the humor message attributes.

In addition to the nuances of the humor response, some of the perspectives on humor such as Tsakona’s (2017) continuum need also to be considered especially when examining the production and reception of humor in various traditional and new media contexts. Our understanding and responses to humor are grounded in our sociocultural context, knowledge resources, and meta-humorous awareness (Tsakona, 2020). The activation of a genre-related schema shapes people’s expectations about the nature of the content to be consumed (e.g., people expect a comedy excerpt to be funny). In assessing responses to humor, the genre-related expectations should therefore be considered as well, either in the form of manipulation check or a statistical control in the subsequent data analysis. To illustrate with these aforementioned nuances in mind, when perceived humor was used to measure humor response, participants can in general agree that the humor stimuli is “funny” or “humorous” due to the genre of the humor content being an excerpt from a comedy show and/or the laughing track embedded in the content, yet considering it as a bad joke, cringy, or inappropriate and disagree with the underlying message.

Moreover, humor responses should be analyzed with other variables of interest in the study as a factor in the model being tested other than simply being treated as a manipulation check measure. To include humor responses variables into the statistical model and hypotheses testing helps improve understanding of the variances explained by the proposed relationships and increase the capability to parcel out the factor attributing to any observed effects.

Based on these reflections, and guided by the results of this review, the following methodological recommendations are offered. First, researchers may consider more humor-theory-guided stimuli development or selection in their experimental manipulations. Working with graphic designers and other media content producers, and utilizing current technological tools, experiments examining more specific component(s) or attribute(s) of a particular type of humor might be devised with conceptual and theoretical guidance. Second, compare different types (techniques) of humor. It was more common to compare a humorous stimulus against a non-humor manipulation. Given the subjective nature of what people find funny, such an approach risks masking nuanced differences. Third, consider more aspects of message attributes that potentially contribute to the effectiveness of humor and include these in the manipulation check or data analysis as controls, if not incorporated into the experimental design. A given humor stimulus may contain incongruities, contextualization cues, an affective tone, it may also be embedded in a genre or exemplifying a specific technique. Putting these into consideration helps improve the understanding of the contribution of each factor into the observed relationships. Fourth, test different dimensions of humor response. Besides humor recognition and appreciation, the comprehension of humor and the agreement with the message underlying such humor are also worth checking. Knowing all four dimensions helps researchers to parcel out the effects of each. Next, measures of humor responses should be incorporated more frequently as an independent or dependent variable. When humor ratings are only used to confirm that a humorous stimuli is rated more humorous than a non-humorous stimuli at the group level, and those humor ratings are not utilized further, the very thing that is often presumed to cause an effect (i.e., humor) is left unspecified.

## 7. Limitations

There are several limitations that should be kept in mind regarding this review. First, there was some difficulty in identifying relevant articles to review. Changes in the media alter the meanings of “media effects.” Accordingly, our interpretation of the term restricting it to experimental designs that permitted quantifying the effect of a media message may seem over restrictive to some. Further, our decision to examine both traditional and social media may appear overly broad to others. Similarly, we used the term “humor” as a broad umbrella term without conducting extensive searches using terms such as “laughter,” “mirth,” or “funniness.” It is therefore possible that some studies were missed as a result.

Second, there were only a small number of articles that met our criteria for review. This meant that both humor and media effects theories that typically rely on surveys, such as uses and gratifications theory, were not represented. By the same token, active researchers who typically employ experimental designs in particular topic areas (e.g., Becker) were over-represented. Finally, there are limitations of the coding itself. Given the range of different topic areas, we decided not to attempt to summarize results. Rather, we were more interested in issues regarding the operationalization of humor and the outcomes associated with those operational decisions. Nevertheless, there were still difficulties in some cases with balancing the trade-offs between the number of categories and the coding of specific items.

## 8. Conclusions

The continued development of humor studies and media effects theories suggests a need for overviews to summarize, not just the results of research (as in meta-analysis), but the processes of research. Based on this review, what is being neglected is attention to basic humor processes, such as mechanisms of incongruities and their resolutions. Thus, we end with a call for greater integration of these two fruitful areas, humor studies and media effects research with the development of theories of humorous media effects.

## Figures and Tables

**Table 1:** Typologies of Humor.

Source	Factor	Component	Description
Buijzen and Valkenburg (2004)	N/A	Slapstick	Physical, pie-in-the-face humor, often degrading
		Surprise	Sudden changes in concepts and images
		Irony	Meaning the opposite of what is being expressed
		Clownish behavior	Exaggerated physical behavior
		Misunderstanding	Misinterpreting a situation
		Parody	Imitating a style or genre
		Satire	Making fun of well-known things, situations, or people
		Miscellaneous	—
van der Wal et al. (2020)	Buffoonery	“Pure” incongruity	Surprising visual, auditory, or conceptual violations of expectation
		Absurdity	Things that go against all logical rules
		Irreverent humor	Humor lacking proper respect for authority or prevailing norms
	Disparagement	Aggressive humor	Ridiculing someone (e.g., discriminatory humor based on sex, race, or intelligence)
		Self-defeating humor	Poking fun at one’s own faults
	Fun	Parody	Poking fun at well-known things, situations, or public figures
		Wordplay	Playing on words, or misinterpreting someone’s words
	Coping with Sexuality	Sexual humor	—
		Coping humor	Using humor to deal with stress and adversities of life
		Slapstick (negative loading)	Physical, pie-in-the-face humor, often involving degradation of status
Ruch et al. (2018)	Dark styles	Irony	Aims at creating a mutual sense of superiority by saying things differently than meant
		Satire	Detects weaknesses, aggressive, but paired with attempts at goodness
		Sarcasm	Aimed at hurting others
		Cynicism	Aimed at devaluing commonly recognized values
	Light styles	Fun	Joking/jesting aimed at spreading good mood and comradeship
		Humor (benevolent)	Aimed at arousing sympathy and understanding of life’s incongruities, imperfections, and one’s own mishaps
		Nonsense	Intellectual and playful; exposes the ridiculousness of sheer sense
		Wit	Quickly reading situations and pointing out non-obvious matters in a funny way

**Table 2:** Eligibility Criteria.

	Inclusion Criteria	Exclusion Criteria
Study design	Original experimental research: Any empirical research examined with any type of between-subject experimental design.	Literature reviews, survey studies, content analysis studies, meta-analyses, systematic reviews, scoping reviews, single-group pre-experimental design, or single-group field experiments.
Context	Context typically studied in media effects research	advertising
Variable of interest	Humorous content as stimulus material(s) in the experimental condition	Humorous content was not used as stimulus material(s) in the experimental condition
Media type	Social media, mainstream media (TV, streaming, newspapers, books), website content, textual content with or without visuals	None
Publication type	Peer-reviewed journal articles	Any other format (e.g., thesis, book)
Publication date	Article: 2008–2025	Any other time frame
Publication language	English	Any other language

**Table 3:** Study features.

Author	Theory/Concept	Msg Attribute	Psychological State (Humor Response)	Humor Usage
Armstrong et al. (2025)	Underdeveloped	Genre and Technique	Recognition	Pretest; MC; Mod Med
Banjo et al. (2017)	Underdeveloped	Context cue	None	None
Becker (2014)	DTH	Genre	PH	DV
Becker (2023)	Cognitive processing	Technique	PH; Recognition (MD)	MC
Becker and Bode (2017)	Cognitive processing	Genre	None	None
Becker and Haller (2014)	Satire types	Genre and Technique	PH; Recognition (MD)	MC; DV
Blanc and Brigaud (2014)	Incongruity-resolution; affective reactions	Genre	PH	Pilot test
Bonnici et al. (2023)	Underdeveloped	Genre	No specific measure *	None; 0
Burmeister and Carels (2020)	Disparagement humor	Technique	None	None; Mod
Cacciatore et al. (2020)	Laughter	Context cue	PH	DV
Davies et al. (2023)	Underdeveloped	Genre	None	Pilot test
Fasoli et al. (2023)	Parody	Genre	PH	Pretest
Futeras and Nan (2017)	E-ELM; counter-arguing	Genre	PH	MC
Greenwood and Gautam (2020)	Disparagement humor	Genre	PH	MC; DV
Heiss (2022)	Cognitive processing	Genre	PH	MC
Jazaieri and Rucker (2025)	Satire	Genre	PH	MC
Jennings et al. (2019)	Cognitive processing; reversal theory	Context cue	None	None
Kim and Vishak (2008)	Cognitive processing	Genre	None	None
Kowalewski (2012)	Cognitive processing	Genre	PH	Pilot test; MC
Martinez and Ramasubramanian (2015)	Underdeveloped	Context cue	PH; enjoyment; stereotyping	Prestet; MC; DV
Matthes and Rauchfleisch (2013)	Parody; message effects	Genre and Technique	PH	MC; Mod Med
Mendiburo-Seguel et al. (2023a)	Disparagement humor; humor styles (corrective and benevolent)	Genre	PH	MC
Mendiburo-Seguel et al. (2023b)	Humor styles (aggressive and self-deprecatory)	Genre and Technique	PH	Pilot test; MC
Mendiburo-Seguel et al. (2024)	Humor and emotions	Genre	PH	None; Mod Med
Moyer-Gusé et al. (2011)	Cognitive processing	Technique	PH	MC
Nabi et al. (2024)	Amusement	Genre	PH	Pilot test; Med
Nabi et al. (2025)	Amusement	Genre	PH	MC; Med
Peifer (2018)	Parody; DTH; HSM	Technique	PH; Agreement	Mod Med
Piaw (2012)	Relief theory; Dual coding; Cognitive transformation	Genre	PH	Pilot test
Prestin and Nabi (2020)	Amusement	Genre	PH	MC; Med
Schmid and Greipl (2025)	BVT; Parody	Genre	PH	Pretest; MC
Skurka et al. (2022)	Parody	Genre	PH	MC
Skurka et al. (2018)	Cognitive processing	Genre	PH	MC; Med
Strick (2021)	Coping mechanism	Genre and Technique	PH	None
Strick et al. (2009)	Cognitive distraction	Genre	None	Pilot test; MC
Waddell and Bailey (2019)	Laugh tracks	Context cue	PH	Med
Warner et al. (2015)	Cognitive processing	Genre	None	None
M. Weber and Quiring (2019)	Laughter	Context cue	PH; EE	Med
S. Weber et al. (2023)	Social identity threat; relief theory; BVT	Genre	PH	Pretest (Study 1); MC (Study 2); Mod
Weinstein et al. (2011)	Hostile humor; superiority theory	Technique	PA	MC; Mixed
Wilson et al. (2025)	Coping humor; relief theory	Technique	PH	Pretest; DV; Med
Yeo et al. (2021)	Incongruity-resolution; encryption model; source likability	Genre and Technique	PH	Med
Yeo et al. (2020)	Incongruity theory	Genre and Technique	PH	Med
Yoon (2019)	Incongruity-resolution	Genre	PH	MC
Young (2008)	Cognitive processing/motivation	Genre	PH; HC	MC
A. L. Zhang and Lu (2022)	Humor types (satire vs. pure humor); Superiority theory; EVT	Genre and Technique	PH	Pretest; MC
B. Zhang (2025)	Disparagement humor; PNT; DTH	Genre and Context cue	Agreement	Mod

Notes. DTH = Disposition theory of humor; E-ELM = Extended elaboration likelihood model; HSM = Heuristic systematic model; BVT = Benign violations theory; EVT = Expectancy violations theory; PNT = Prejudiced norm theory; PH = Perceived Humor; MD = Message discounting; HC = humor comprehension; EE= emotional expression; MC = manipulation check; Med = mediation; Mod = moderation. * Bonnici et al. (2023) included a “laugh” emoji as part of a selection of emojis used as a composite variable.

## Data Availability

No new data were created or analyzed in this study. Data sharing is not applicable.

## Note

1 Coding message discounting as a measure of humor recognition is based on the content of the items. For example, the items “The author of the message was joking” asks participants to evaluate whether or not the message was an example of humor. The term “message discounting” is tied to a particular cognitive processing explanation that contrasts the nonseriousness of humor with seriousness of some alternative message form/content.
